# Behavioral and Biochemical Effects of KXS on Postmyocardial Infarction Depression

**DOI:** 10.3389/fphar.2020.561817

**Published:** 2020-08-27

**Authors:** Yuan Hu, Xu Liu, Tianyi Zhang, Chao Chen, Xianzhe Dong, Yan Can, Ping Liu

**Affiliations:** ^1^Medical Supplier Center, Department of Pharmacy, PLA General Hospital, Beijing, China; ^2^Savaid Medical School, University of Chinese Academy of Sciences, Beijing, China; ^3^Department of Basic Theory of Chinese Medicine, School of Pre-clinical Medicine, Guangzhou University of Chinese Medicine, Higher Education Mega Center, Guangzhou, China; ^4^The Research Centre of Basic Integrative Medicine, Guangzhou University of Chinese Medicine, Higher Education Mega Center, Guangzhou, China

**Keywords:** Kai-Xin-San, cardioprotective effect, antidepressive effect, depression, coronary heart disease

## Abstract

**Background:**

Depression and coronary heart disease (CHD) often occur together in clinical practice. As a traditional Chinese medicine, Kai-Xin-San (KXS) has been widely used for the treatment of emotion-related disorders. In the present study, we aimed to explore whether KXS had both antidepressive effects and cardioprotective functions in a rat model of myocardial ischemia (MI) with depression.

**Methods:**

A total of 50 SD rats were randomly assigned into five groups as follows: normal control (control group), celiac injection of isopropyl adrenaline (ISO) (MI group), depression (depression group), MI+ depression (model group) and MI+ depression treated with intragastric administration of 370 mg/kg KXS (KXS group). MI was induced by subcutaneous injection of 85 mg/kg ISO. Depression was developed by a 7-week chronic mild stress (CMS) challenge. Behavioral test was conducted before and during the experiment. Echocardiography and biochemical analysis were carried out after 7 weeks of CMS challenge.

**Results:**

After 7 weeks of experiment, depression-like behaviors were observed in all the groups except for control and KXS groups, and KXS treatment dramatically increased open-field test scores and sucrose consumption (P < 0.01 vs. model group). Echocardiography and biochemical analysis showed that KXS treatment could improve levels of ejection fraction (EF) and fractional shortening (FS), which were reduced by depression and ISO challenge. Meanwhile, KXS treatment significantly decreased the levels of creation kinase MB (CK-MB) and lactate dehydrogenase (LDH), which were increased in the model group. The activities of superoxide dismutase (SOD), glutathione peroxidase (GSH-PX), catalase (CAT) were increased, while the malondialdehyde (MDA) activity was significantly decreased in the KXS group. Moreover, KXS treatment reduced the levels of C-reactive protein (CRP), interleukin-6 (IL-6) and tumor necrosis factor-α (TNF-α) in myocardial tissue compared with the model group.

**Conclusions:**

KXS had antidepressant-like activity and offered cardioprotective effects against ISO-induced myocardial infarction with depression.

## Background

Depression and coronary heart disease (CHD) are both common conditions and often occur together. There is a complex inter-relationship between depression and CHD, where depressive illness results in cardiovascular disease and in turn heart disease causes depression (Goldston and Baillie, 2008; Seymour and Benning, 2009). Increasing evidence has shown that depression can be linked to an increased risk of CHD in an independent manner, and patients with CHD and depression exhibit a higher morbidity and mortality compared with those with CHD only (Nemeroff and Goldschmidt-Clermont, 2012; Headrick et al., 2017). As the classical emergent manifestation of CHD, acute myocardial ischemia (MI) is associated with an increased mortality risk (Wang et al., 2014). Depression is an important clinical issue in MI patients due to its extreme commonness, and the comorbidity complicates the depression treatment and worsens the cardiovascular prognosis (Shapiro, 2015). Although many therapeutic strategies have been used to treat CHD or MI patients with depression (Colquhoun et al., 2013), such as psychological therapy (cognitive-behavioral therapy and interpersonal psychotherapy) and pharmacological therapy (fluoxetine, sertraline, citalopram, and mirtazapine), clinical outcomes remain unsatisfied, which can be mainly attributed to the unavoidable adverse effects of these treatments. Therefore, a great deal of efforts have been made to develop efficient therapies based on traditional Chinese medicine (TCM), which is a crucial part of complementary and alternative medicines (Francis et al., 2015), and in some parts, its multi-components with the multi-target characteristics may contribute to complex somatopsychic illness (Wang et al., 2012). The TCM Kai-Xin-San (KXS) is composed of ginseng (Panax ginseng C.A. Meyer), hoelen (Poria cocos F.A. Wolf), polygala (Polygala tenuifolia Willd), and acorus (Acorus calamus var. angustatus Besser syn. Acorus tatarinowii Schott) with a ratio of 3:3:2:2 (Hu et al., 2013a; Hu et al., 2013b), which was first recorded in Thousand Formulae for Emergency (Bei Ji Qian Jin Yao Fang) by Sun Si Miao. This formula is widely used in Asia, especially in China, and KXS is effective in treating depression-associated deficits of learning and memory. In our previous studies, we have proved its antidepressive effect using different animal models, such as the chronic mild stress (CMS) challenge, tail-suspension test, and forced swim test (Cao et al., 2012; Zhou et al., 2012; Dong et al., 2013). KXS is effective in improving behavior disorders by activating TrkB/ERK/CREB/BDNF and TrkB/PI3 K/CREB/BDNF pathways and influencing various inflammatory pathways, such as IL-6 and TNF-α (Dong et al., 2016). In the present study, we further explored Shen-Zhi-Ling (SZL) tablets, which are the proprietary Chinese medicines based on KXS decoction, and verified its safety and efficacy in patients with depression (unpublished data). Huang YF (Huang et al., 2000) et al. have found the protective effect of KXS on kidney in rats using ischemia-reperfusion injury model. KXS can increase SOD and NO activities, decrease MDA content and improve kidney tissues. These finding suggest that KXS can protect the blood vessels effectively. Here, we hypothesized that KXS might balance psychosomatic status and effectively relieve symptoms of CHD with depression. In this study, we investigated whether KXS possessed both antidepressive effects and cardioprotective functions.

## Methods

### Preparation of KXS

KXS, supplied in the form of a powdered herbal extract, was obtained from Beijing Tongrentang Drug Store (Beijing, China). All KXS formulations were provided by the Chengdu Luye Medicinal Material Plantation Co., Chengdu, China. The voucher specimens of the four plants, identified by Professor Ping Liu and registered under the numbers NU−90111, NU−82003, NU−79015 and NU−80617, respectively, were preserved at the pharmacy of the Herbarium of Traditional Chinese Medicinal (TCM), Chinese People’s Liberation Army (PLA) General Hospital (Beijing, China). The total extract was prepared as previously reported (Cao et al., 2012). Averagely, 1 g yielded KXS powder equaled to 4.83 g total herbs. The standardization of KXS was ensured by determining its chemical fingerprinting according to our previous study (Hu Y. et al., 2014). Thin-layer chromatography (TLC) of the total extract and each herb showed in **supplement 1**.

### Animals

**Figure 1:**
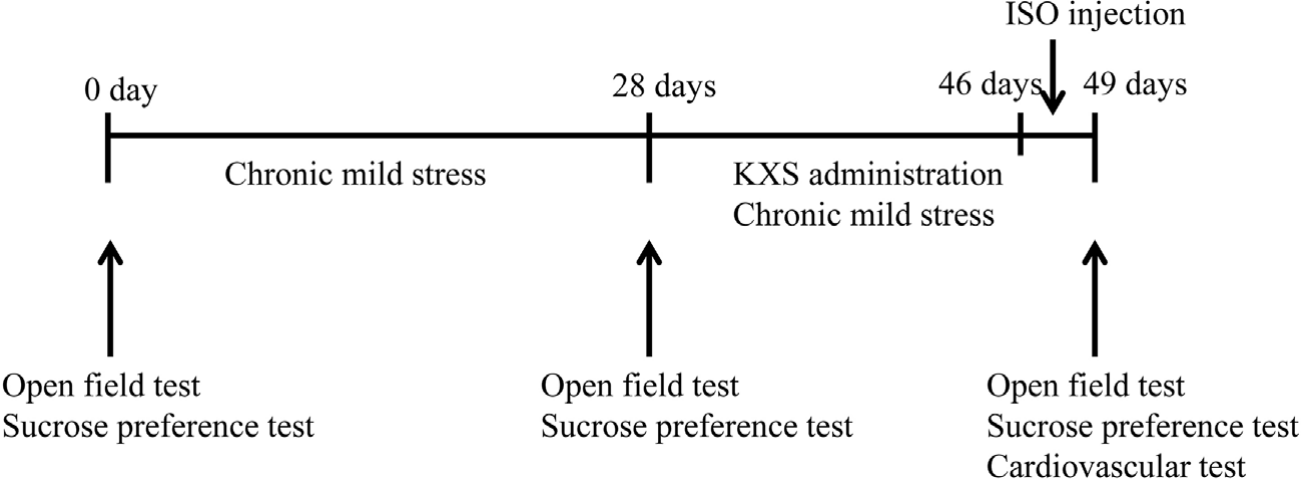
Experimental protocol of timeline depicting the behavioral adaptation and the behavioral tests. ISO, isopropyl adrenaline; KXS, Kai-Xin-San.

Male Sprague-Dawley (SD) rats, weighing 250–270 g, were used for the experimental procedures. Rats were purchased from Beijing Vital River Laboratory Animal Technology Co., Ltd. and given 3 days to acclimate to the housing facility. Animals were housed in 475 × 345 × 200 mm cages and given free access to food and water. A total of 50 SD rats were randomly and evenly divided into five groups (n=10) as follows: normal control (control group), isopropyl adrenaline (ISO) model (MI group), CMS model (depression group), MI+ depression (model group) and MI+ depression treated with intragastric administration of 1,785 mg total herbs/kg KXS (about 370 mg/kg KXS, KXS group), and the antidepressive effect of KXS at such dosage has been proved in several previous studies (Hu et al., 2013a; Dong et al., 2016). Except for the KXS group, other groups were given the same volume of saline by intragastric administration. Rats in all other groups were challenged with the same CMS for 49 days, except for the control group. Behavioral tests, including open-field and sucrose preference tests, were performed during the experiment. For the KXS group, rats were administered by gavage with KXS dissolved in distilled water once a day for 21 days after 28 days of CMS challenge, and meanwhile, the rats were continuously challenged with CMS. In order to establish MI and MI+ depression models, rats were fixed in the supine position, and 85 mg/kg ISO (Sigma-Aldrich, St. Louis, MO, USA) was subcutaneously injected on the limb roots and back in three consecutive days (Cheng et al., 2013). The MI model was verified by echocardiography as well as the levels of serum creatine kinase MB (CK-MB) and lactate dehydrogenase (LDH). All animal-related experiments were approved by the Animal Experimentation Ethics Committee of Chinese PLA General Hospital (number: 2017-x15-23). At the end of the trial, the rats were sacrificed by cervical dislocation. **Figure 1** illustrates the protocol of the animal experiment. At day 49, the rats were anesthetized rats (10% chloral hydrate, 350 mg/kg) and sacrificed by cervical dislocation. Rats no exhibited signs of peritonitis after the administration of 10% chloral hydrate.

### CMS Model

In the present study, the CMS model was employed as a validated animal model of depression (Wang et al.). All rats were challenged with the following stressors (Hu et al., 2013a), including repeated confinement to a small (38 × 20 × 16 cm) cage, restraint (1 h), water deprivation (24 h), food deprivation (24 h), isolation (24 h), flashing light (3 h), forced cold-water swimming (10 min) or group-housed in a soiled cage overnight. Above-mentioned stressors were given every day for 49 days in a random and unpredictable order.

### Open-Field Test and Sucrose Consumption Test

The open-field test (Yan et al.) and sucrose consumption test (Dang et al., 2009) were employed to assess the behavior and anhedonic-like state of all rats. These tests were performed on day 0, 28 and 49 during the experiment. For open-field test, the emotional state, including assessment of horizontal movements (the total number of crossing squares) and vertical movements (grooming and rearing), during a period of 5 min was recorded as the behavioral response to a new environment. A special white square consisting of 25 sectors with black stripes on the ground was used (80 × 80 × 40 cm) in the test. Animals were individually placed in the same central sector, and a video camera was used to record their activity during a period of 5 min. Subsequently, the results of video tapes were analyzed by observers (ANYmaze, Stoelting Co., USA).

Sucrose consumption test is a trial reflecting the anhedonic-like state of animals. Rats were deprived of food and water for 24 h prior to sucrose consumption test, and then they were fed with two pre-weighted bottles containing either 1% sucrose solution or water for 1 h. Water intake was determined by weighing the bottles before and after each test. All tests were carried out in the home cage, by which the extraneous novelty and disturbance were minimized. The sucrose preference was calculated as sucrose intake/total water intake (sucrose intake+water intake)(Chen et al.).

### Echocardiographic Determination

The hairs on the chest of anesthetized rats were removed with depilatory paste (Strep Co., Milan, Italy). Subsequently, rats were placed on the scanning platform of a high-resolution ultrasound system in the supine position (VisualSonics Corporation Vero 770, Canada). The chest of each rat was covered with a layer of ultrasonic coupling agent. M-mode echocardiography was conducted to determine cardiac parameters, including length of left ventricular internal diameter at end-diastole (LVIDd), length of left ventricular internal diameter at end-systole (LVIDs), ratio of stoke volume at left ventricular end diastolic volume (ejection fraction, EF), and (LVIDd-LVIDs)/LVIDs (fractional shortening, FS)

### Measurement of Cardiac Marker Enzymes

After the ECG detection, arterial blood samples were collected and centrifuged at 3,000 rpm for 15 min, and serum was collected and then stored at −80°C. Serum samples were thawed at room temperature before analysis. The serum levels of CK-MB and LDH were determined by commercial kits (Nanjing Jiancheng Bioengineering Institute, Nanjing, China) to evaluate the myocardial damage according to the manufacturer’s instructions.

### Measurement of Antioxidant Enzymes and Inflammation Assay

Activities of SOD, MDA, CAT, and GSH-Px in myocardial tissue were determined using commercial kits (Nanjing Jiancheng Bioengineering Institute, Nanjing, China) according to the manufacturer’s instructions. Levels of CRP, IL-6 and TNF-α in myocardial tissue were determined by ELISA kits using specific antibodies (Antix Biotech China Co., Ltd., Changsha, China) according to the manufacturer’s instructions. Absorbance at a wavelength of 450 nm was determined on a microtiter plate reader (PerkinElmer, Inc., Waltham, MA, USA), and results were expressed as ng/L.

### Histological Studies

The freshly dissected heart tissues were fixed in 10% formalin solution. The sectioning and staining of heart tissue were performed according to previously established protocols. Sections (5 mm, Leica RM 2125, Germany) from the left ventricle were stained with hematoxylin and eosin (H&E) and examined by light microscopy (Nikon, Tokyo, Japan) at a magnification of 200 ×.

### Statistical Analysis

Results were expressed as means ± SD. Statistical analysis was performed using the one-way ANOVA, followed by Dunnett’s t test. P < 0.05 was considered as statistically significant.

## Results

### Behavioral Analysis

**Table 1** displays the fluid intake during the sucrose consumption test and the changes of open-field test scores, which were used to define anhedonia in five experimental groups. There were no differences in sucrose intake test and open-field test scores among various groups at the baseline. However, after 28 days, the sucrose consumption and open-field test scores were significantly reduced in the depression and model groups (data not shown), and such results were consistent with previous study (Dong et al., 2016). After 49 days, the sucrose consumption and open-field test scores were significantly reduced in the MI group, depression group, and model group. For these three injured groups, the levels of sucrose consumption and open-field test score were similar between the model and depression groups, while higher levels were observed in the MI group. Collectively, KXS administration could significantly improve sucrose consumption and open-field test scores (vs. model, P < 0.01).

**Table 1:** The comparison of the open-field test scores and sucrose consumption of rats in different groups after experiment.

Group	Open field test			Sucrose consumption (%)
	Horizontal movement (scores)	Vertical movement (scores)	Total (scores)	
Control	97.9 ± 16.02	24.50 ± 6.95	122.40 ± 21.13	0.81 ± 0.04
IM	80.25 ± 7.05^##^	15.38 ± 3.97^##^	95.63 ± 8.25^##^	0.72 ± 0.06^#^
Depression	36.25 ± 8.57^##^	8.00 ± 3.26^##^	44.25 ± 10.04^##^	0.46 ± 0.10^##^
Model	39.34 ± 7.64^##^	9.34 ± 2.50^##^	48.67 ± 6.93^##^	0.46 ± 0.14^##^
KXS	67.25 ± 5.58^**^	13.75 ± 2.06^**^	81.00 ± 5.98^**^	0.68 ± 0.08^**^

Compared with control, ^#^P < 0.05, ^##^P < 0.01; Compared with model, ^**^P < 0.01.

Control, normal rats, IM, injected with ISO; depression, chronic mild stress rats; model, depression with ISO; KXS, KXS+ depression with ISO.

### The Effect of KXS on Myocardial Injury

**Figure 2:**
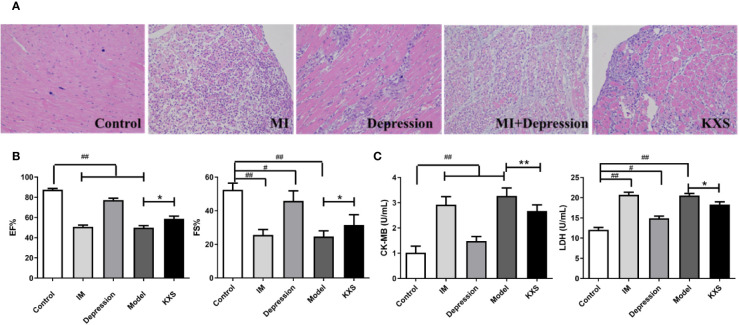
The cardioprotective effect of KXS. Rats were randomly assigned into five groups: control, MI, depression, model and KXS. After experiment, the myocardial injury was determined by HE staining, echocardiography (EF and FS) and cardiac marker enzymes (CK-MB and LDH). **(A)** Representative HE staining of myocardial tissue (×200). **(B)** The EF and FS changes in groups. **(C)** The CK-MB and LDH changes in groups. Compared with control group, ^#^P < 0.05, ^##^P < 0.01; Compared with model group, *P < 0.05, **P < 0.01. EF, ejection fraction; FS, fractional shortening; CK-MB, serum creatine kinase MB; LDH, lactate dehydrogenase; ISO, isopropyl adrenaline; Control, normal rats; IM, injected ISO; Depression, chronic mild stress rats; Model, depression with ISO; KXS, KXS+ depression with ISO.

**Figure 2A** shows that the myocardial cells exhibited uniform HE staining, and striated filaments of cardiac myocytes were neatly arranged with clear cell boundaries. In the control group, typical normal cardiac myocytes were observed. Only minor infiltration of inflammatory cells was detected, and no apparent fibrosis was found. Compared with the control group, fibrotic tissues were slightly increased, and the gaps of myocardial fibers became wider in the depression group. In the MI and model groups, myocardial fibers were thicker and disordered, the gaps were wider, and extensive cardiomyocyte injury and necrosis were observed, indicating increased infiltration of inflammatory cells. Compared with the model group, less infiltration of inflammatory cells was observed in the KXS group, fibrous tissues were significantly decreased, and only fewer disordered cells were detected. **Figure 2B** reveals that EF and FS were decreased in the MI group, depression group, and model group compared with the control group (P < 0.01 or P < 0.05). Compared with the model group, levels of EF and FS were significantly increased in the KXS group (P < 0.05). **Figure 2C** shows that the levels of CK-MB and LDH were higher in the MI group, depression group, and model group compared with the control group (P < 0.01 or P<0.05). Moreover, the levels of CK-MB and LDH were significantly decreased in the KXS group compared with the model group (P < 0.01 or P<0.05).

### The Effect of KXS on Antioxidant Enzymes and Inflammation Index

**Figure 3:**
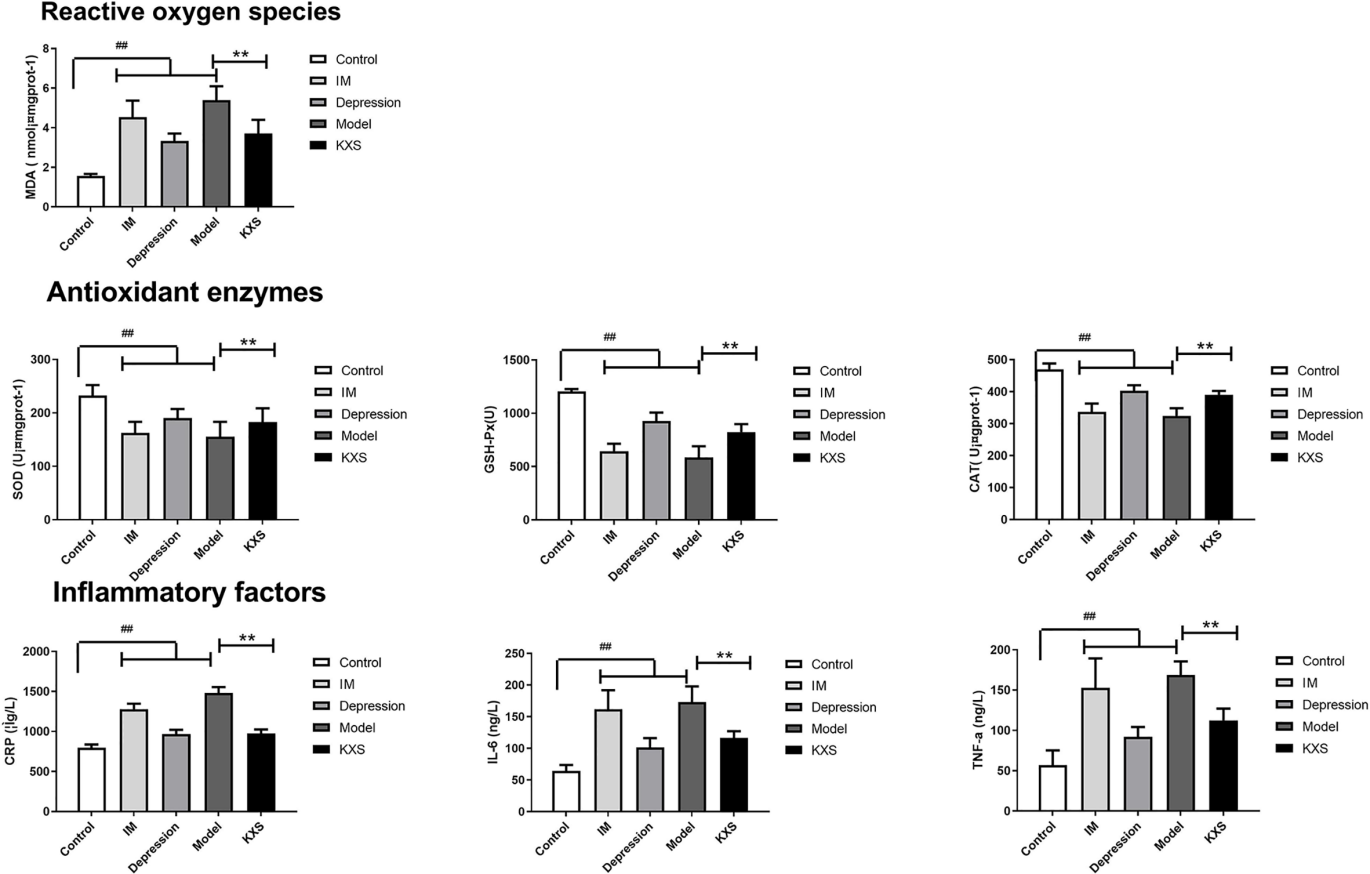
The comparison of antioxidant enzymes and inflammatory factors in myocardial tissue in different groups after experiment. Rats were randomly assigned into five groups: control, MI, depression, model and KXS. After experiment, reactive oxygen species (MDA), antioxidant enzymes (SOD and GSH-Px) and inflammatory factors (CRP, IL-6, and TNF-a) in myocardial tissue were determined. Compared with control group, ^##^P < 0.01; Compared with model group, **P < 0.01. MDA, malondialdehyde; SOD, superoxide dismutase; GSH-Px, glutathione peroxidase; CRP, C-reactive protein; IL-6, interleukin-6; TNF-α, tumor necrosis factor-α; control, normal rats, IM: injected with ISO, Depression: chronic mild stress rats, Model: depression with ISO, KXS: KXS+ depression with ISO.

**Figure 3** indicates that the MDA activity was significantly increased, while the activities of SOD, CAT, and GSH-Px were significantly decreased in other groups compared with the control group (P < 0.01). Moreover, the highest MDA activity was found in the model group. Compared with the control group, the levels of CRP, IL-6 and TNF-α were significantly increased in other groups (P < 0.01). The activities of antioxidant enzymes and levels of inflammatory factors were similar between the MI and model groups. Compared with these two groups, higher levels of SOD, CAT, and GSH-Px but lower levels of CRP, IL-6 and TNF-α were observed in the depression group. After KXS treatment, the activity of MDA was significantly decreased (P < 0.01), while the activities of SOD, CAT, and GSH-Px were significantly increased compared with the model group (P < 0.01). In addition, the levels of CRP, IL-6 and TNF-α were significantly decreased in the KXS group compared with the model group (P < 0.01).

## Discussion

Generally speaking, comorbid depression occurs in 9.3-23% of individuals with one or more chronic physical diseases (Moussavi et al., 2007). Similarly, the prevalence of depressive disorder is 15%–30% in MI patients, which is about five times higher compared with the general population. A meta-analysis indicates that the risk of cardiac mortality and new cardiovascular events is increased by 2.4-fold and 2-fold under the conditions of post-MI depression, respectively (Meijer et al., 2013). In the present study, lower levels of EF and FS but higher levels of CK-MB and LDH were observed compared with normal rats, indicating the myocardial injury in depressive rats. Moreover, rats in the MI group showed depression-like behaviors. These results verified the relationship between depression and MI. Although many therapeutic strategies have been used to treat MI, the outcomes remain unsatisfied. Therefore, it is urgently necessary to develop an alternative medicine for MI treatment and to investigate its potential mechanism.

Our previous study has shown the anti-depressive effect of KXS in several depression models (Dang et al., 2009; Yan et al., 2016). In this study, in order to investigate whether KXS possessed both antidepressive effects and cardioprotective functions, we first established a rat model of MI with depression. The model was established using CMS-challenged rats, followed by ISO-triggered induction of MI. ISO is a synthetic catecholamine and β-adrenergic agonist, and it triggers necrosis, hypoxia, hyperplasia, and MI at high doses (Abbas, 2016; Zhang et al., 2017). In our current study, MI model was established in rats by intraperitoneal administration of ISO for three successive days with a dose of 85 mg/kg and a 24-h interval between the applications. Then the model was tested by depression-like behavior and myocardial injury. For the determination of depression-like behavior, we found that sucrose intake and open-field test scores were significantly reduced in rats exposed to CMS. For myocardial injury, we tested it by echocardiography and cardiac markers. MI leads to significantly decreased contractile and diastolic forces of the heart, especially in the region of the MI. These alterations can be attributed to ischemia in the ventricular walls, limiting the movement. Previous studies have reported that lesion severity and left ventricular function determine the prognosis of MI. Therefore, left ventricular function plays a crucial role in evaluating treatment effect (Wang et al., 2015). Echocardiography is a useful tool to determine the cardiac function and assess the changes in left ventricular function by EF and FS, which can accurately diagnose MI in animals (Xu et al., 2018). CK-MB and LDH, related to the cellular damage and loss of functional integrity, are important myocardial enzymes in the evaluation of myocardial injury and congestive heart failure, and these markers have also been used to diagnose MI (Dhivya et al., 2017; Lin et al., 2017). In this work, the levels of EF and FS were significantly decreased, while the levels of plasma CK-MB and LDH were increased in the MI and model groups compared with the control group. The above-mentioned findings indicated that an MI+ depression model was successfully established. In addition, such model showed similar results to those of the depression group in behavioral test, including open-field test and sucrose consumption. Moreover, the MI+ depression model also exhibited similar results to those of the IM group in terms of echocardiography (EF and FS), cardiac markers (CK-MB and LDH), antioxidants (SOD, GSH-Px, and CAT) and inflammatory factors (CRP, IL-6, and TNF-α).

After KXS treatment, open-field test scores were increased, and the sucrose consumption was also elevated, suggesting the antidepressive effects of KXS, that consisted with our previous studies (Hu et al., 2011; Dong et al., 2016). KXS could partially increase the levels of EF and FS and decrease the serum levels of CK-MB and LDH, indicating that KXS could not only improve the depression-like behavior of rats in the model group but also improve the cell functional integrity and restrict the leakage of these enzymes into circulation. In addition, HE staining is another method frequently used in evaluation of myocardial injury (Zhang et al., 2014; Lin et al., 2017). In the current study, the cardioprotective effects of KXS were further validated by the significantly improved histological results. The histopathological changes were significantly restored by KXS treatment, and less area of degeneration was found in the heart tissue sections of the KXS group compared with the model group. These findings also provided convincing evidence that KXS exerted cardioprotective effects against ISO-induced myocardial injury.

According to published many literatures about KXS antidepressant mechanism, such as Dong XZ (Dong et al., 2017) and Wang S (Wang et al., 2016). Therefore, we believed antioxidant enzymes and inflammation index are related with depression. At the same time, antioxidant enzymes and inflammation index are more related with CHD, so we hypothesized the antioxidant enzymes and inflammation index might be the co-morbid condition of heart disease and depression. The status of oxidative stress was assessed by determining the MDA activity and activities of several endogenous antioxidants. MDA [a reactive oxygen species (ROS)] as well as SOD, GSH, and CAT (antioxidants) are important markers of myocardial oxidative damages (Hu X. et al., 2014; Yang et al., 2014). Antioxidants can scavenge free radicals under pathologic conditions, and excessive ROS disturbs the existing balance of antioxidant system (Hu et al., 2016). KXS treatment reduced the MDA activity, and the activities of SOD, GSH, and CAT were restored by KXS treatment compared with the MI rats. The over-production of pro-inflammatory cytokines, including IL-6, CRP, and TNF-α, can be induced by excessive oxidative stress. Many studies have linked the elevated levels of inflammatory markers to ischemia (Willerson and Ridker, 2004). KXS treatment decreased the contents of pro-inflammatory cytokines, suggesting that its cardioprotective effects were partially attributed to its anti-inflammatory properties. These findings were consistent with previous data that KXS treatment can modulate the disturbance of cytokines induced by chronic fatigue syndrome (Cao et al., 2012). In addition, these results were consistent with the histological evaluation of inflammatory cellular infiltration in myocardial tissues.

A randomized, double-blind, positive-drug, parallel-controlled clinical trial has been conducted to evaluate the efficacy and safety of KXS in treatment of mild/moderate depression since 2015. Pharmacologically, KXS with its active compounds may exert its effects on mental illness mainly through adjusting the adrenergic activation, HPA axis dysfunction and immuno-inflammation (Hu et al., 2011; Cao et al., 2012; Hu et al., 2013a), which are the co-mechanisms in psycho-cardiology (Headrick et al., 2017). In the present study, we demonstrated that KXS had beneficial effects on cardiac function in MI rats. Moreover, KXS treatment showed remarkable anti-depression and cardioprotective effects in an MI rat model with depression. The cardioprotective function of KXS might be associated with its ability to attenuate oxidative stress, leading to reduced myocardial damage. Taken together, our findings supported that KXS had therapeutic effects on patients with cardiovascular disease. However, such beneficial effects of KXS were only verified by behavior and biochemical tests. Therefore, further studies are required to reveal its underlying mechanisms.

In the present study, we found that KXS had antidepressive effect and cardioprotective function by a rat model of MI with depression. After KXS treatment, open-field test scores, and sucrose consumption, which are indicators for evaluating depression-like behaviors, were significantly increased compared with the model group. For cardioprotective effects, KXS treatment could improve the EF and FS, down-regulate CK-MB, LDH, and MDA, and up-regulate SOD, GSH-PX, and CAT. Meanwhile, KXS treatment reduced the levels of CRP, IL-6, and TNF-α in myocardial tissue compared with the model group.

## Data Availability Statement

The raw data supporting the conclusions of this article will be made available by the authors, without undue reservation, to any qualified researcher.

## Ethics Statement

All animal related experiments were approved by the Animal Experimentation Ethics Committee of Chinese PLA General Hospital (number: 2017-x15-23). All animal handling procedures were carried out in compliance with the ‘Principles of Laboratory Animal Care’ and the Chinese legislation for the use and care of laboratory animals.

## Author Contributions

YH, XL, PL, and YC contributed to study design, trial, and writing of this study. TYZ, CC, and XZD contributed to the trial.

## Funding

Financial support was provided by National Natural Science Foundation of China (No 81573876 and 81973520) for the study and collection, analysis, interpretation of data and in writing the manuscript, and Clinical Research Support Funding of PLA General Hospital (2016FS-TSYS-2045) for the study and analysis.

## Conflict of Interest

The authors declare that the research was conducted in the absence of any commercial or financial relationships that could be construed as a potential conflict of interest.

## Supplementary Material

Supplement Figure 1 Thin-layer chromatography (TLC) of the total extract and each herb.
