# Supplementary figures and images for: Behavioral and Biochemical Effects of KXS on Postmyocardial Infarction Depression
Source: Front Pharmacol. 2020 Aug 27;11:561817. doi: 10.3389/fphar.2020.561817 (PMC7481476; doi:10.3389/fphar.2020.561817)

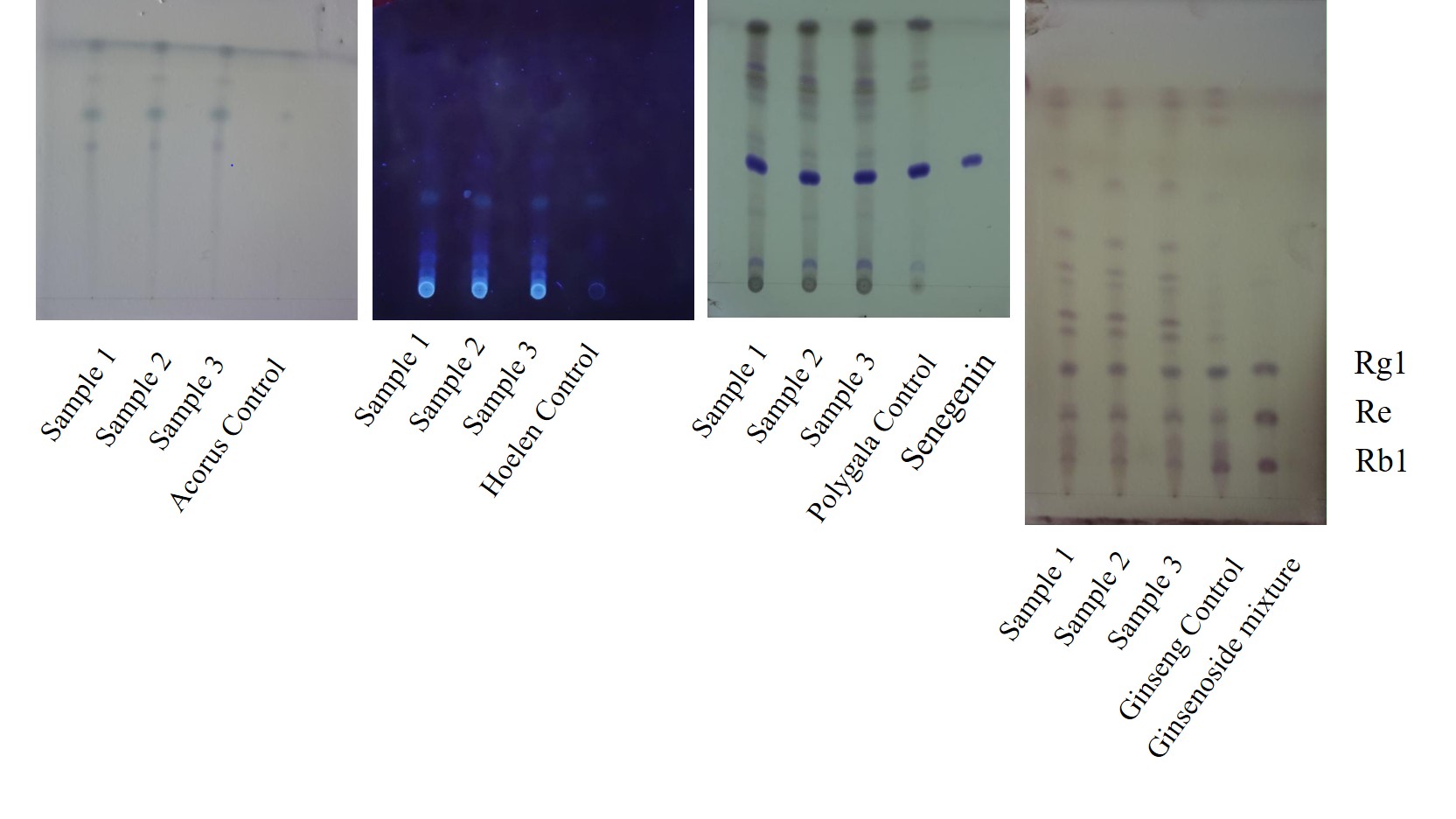

Supplement: Supplement Figure 1 — Thin-layer chromatography (TLC) of the total extract and each herb. [file Image_1.jpeg]
